# Extended Preservation of Heart Grafts: LYPS Solution Maintains Cardiac Function During 20-Hour Static Cold Storage

**DOI:** 10.3390/ijms262211170

**Published:** 2025-11-19

**Authors:** Marie Védère, Evan Faure, Christophe Chouabe, Lionel Augeul, Ninon Cadot-Jet, Georges Christé, Yanis Charouit, Mégane Lo Grasso, Alexandre Ravon, Régine Cartier, Gabriel Bidaux, René Ferrera, Hala Guedouari, Delphine Baetz

**Affiliations:** 1 CARMEN Laboratory, INSERM, INRAE, Université Claude Bernard Lyon 1, University of Lyon, 69500 Lyon, France; marie.vedere@univ-tlse3.fr (M.V.); evan.faure@etu.univ-lyon1.fr (E.F.); christophe.chouabe@univ-lyon1.fr (C.C.); lionel.augeul@univ-lyon1.fr (L.A.); ninon.cadot-jet@univ-lyon1.fr (N.C.-J.); christe.georges@laposte.net (G.C.); yanis.charouit@univ-lyon1.fr (Y.C.); meg69580@gmail.com (M.L.G.); alexandre.ravon@univ-lyon1.fr (A.R.); gabriel.bidaux@univ-lyon1.fr (G.B.); rene.ferrera@univ-lyon1.fr (R.F.); delphine.baetz@univ-lyon1.fr (D.B.); 2 Groupement Hospitalier Est, Hospices Civils of Lyon, 69500 Bron, France; regine.cartier@chu-lyon.fr

**Keywords:** heart transplantation, ischemia–reperfusion injury, static cold storage

## Abstract

Heart transplantation is severely limited by the shortage of suitable donor grafts, partly due to myocardial vulnerability to ischemia–reperfusion injury and the lack of standardized preservation strategies. Current solutions only partially maintain myocardial viability, compromising post-transplant function. To address this issue, we made further improvements to our preservation solution, LYPS (Lyon Preservation Solution), based on mitochondrial metabolic activation and the limitation of membrane depolarization. We first evaluated commonly used extracellular solutions (Celsior and St. Thomas (ST)) on cardiac cell lines (H9C2) exposed to 20 h of cold (4 °C) simulated ischemia followed by 2 h of simulated reperfusion. In parallel, the same three solutions were compared in isolated pig hearts subjected to 20 h of cold static storage followed by reperfusion, with a group directly reperfused with blood at 37 °C serving as the control. Heart function was assessed using a non-working heart preparation, while mitochondrial functions and electrophysiological analysis were evaluated via biopsies and isolated cardiomyocytes. LYPS provided superior protection against cell death and mitochondrial membrane potential loss in vitro, outperformed ST in preserving mitochondrial function, and limited troponin I release by the heart. During reperfusion, LYPS-treated hearts showed improved functional recovery and contractility and better rhythmicity with almost no defibrillation requirements. These effects may involve the modulation of the repolarizing IK_1_ current. Overall, LYPS effectively preserves myocardial viability and function, representing a promising strategy to enhance graft quality during long-term cold preservation, even through using cold static storage.

## 1. Introduction

Heart transplantation remains the most effective treatment for patients with advanced heart failure, as it significantly improves survival and quality of life [1]. Despite advances in surgical techniques, perioperative care, and immunosuppression, critical challenges persist, particularly for transplantation and long-term graft function. The number of cardiac transplants reached a plateau in the mid-1990s due to a shortage of donor hearts, while the waiting list continued to increase [2]. To address this, donor eligibility criteria have been expanded to include older hearts and organs harvested from greater distances, which results in longer cold ischemic times. However, prolonged cold ischemia is not recommended, as it may undermine graft quality and patient outcomes. Retrospective studies have shown that the duration of cold ischemia is a reliable predictor of both 5-year survival and post-transplant mortality [3]. Indeed, Tang et al. reported that ischemic times exceeding 3 h were associated with higher mortality compared to shorter periods [4]. Therefore, effective preservation strategies are crucial to maintain myocardial viability during transport, to minimize ischemia–reperfusion injury, and to optimize post-transplant outcomes, particularly when cold ischemic times are extended. To this aim, two main strategies have been considered: (i) replacing static cold storage (SCS) with dynamic machine perfusion (MP) and/or (ii) optimizing the composition of the preservation solution. Although MP can provide continuous perfusion and metabolic support, it is a complex approach involving bulky, difficult-to-transport devices, an increased risk of infection, the requirement for specialized personnel, and high costs [5,6]. Moreover, randomized controlled trials have not yet demonstrated a clear advantage of MP over conventional SCS, and its clinical benefit is still debated [7,8]. In this context, improving the preservation solution represents a practical and less restrictive approach, offering the potential to improve graft protection and outcomes during hypothermic storage.

Although more than one hundred preservation solutions, both experimental and clinical, have been proposed for static hypothermia [9], none have gained universal consensus. Several are currently used in routine clinical use, including the University of Wisconsin (UW), Celsior, Saint Thomas, and Custodiol (HTK) [10,11]. These solutions are formulated to reduce metabolic demand, maintain ionic balance, and limit cellular injury during cold storage. However, their efficacy in heart transplantation remains debated, as none provides complete protection against ischemia–reperfusion injury. In this context, the development of novel preservation solutions with enhanced cardioprotective properties represents a promising strategy to improve graft quality. To address this gap, our team was the first to develop an original solution, LYPS (Lyon Preservative Solution), with an innovative composition [12,13,14,15]. It is the combination of different compounds in a mixture that gives LYPS its originality by providing increased protection against the harmful effects of cold ischemia–reperfusion (Table S1). Each component of LYPS was assessed to create an efficacious solution based on four guiding principles: (i) the activation of cellular metabolism at low temperatures, (ii) protection of mitochondria against reperfusion injury, (iii) enhancement of cell survival and inhibition of death pathways, and (iv) prevention of primary graft dysfunction at reperfusion. The objective of the present study was to evaluate the effectiveness of LYPS in maintaining myocardial integrity during extended static cold storage, in comparison with standard clinical preservation solutions, using both rat cardiomyoblasts and an ex vivo isolated pig heart model.

## 2. Results

### 2.1. Protective Effect of LYPS on H9C2 Cardiac Cells Subjected to In Vitro Simulated Cold Ischemia–Reperfusion

**Figure 1:**
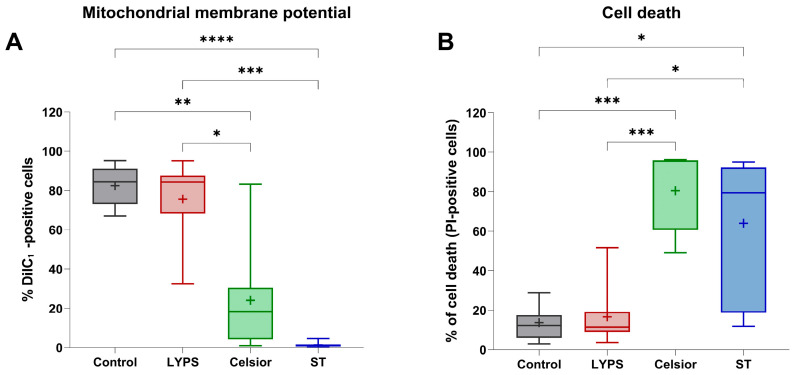
The effect of LYPS solution on H9C2 cell integrity at 4 °C. H9C2 cells were preserved for 20 h at 4 °C in the different tested solutions (LYPS, Celsior or St. Thomas (ST)), followed by 2 h of reperfusion at 37 °C in DMEM complete medium. Control cells were maintained for 22 h in complete medium. Mitochondrial membrane potential was assessed by flow cytometry using 2 nM DiIC_1_ probe (**A**), and cell death was quantified as the percentage of PI-positive cells (**B**). For each independent experiment (n ≥ 7), multiple technical replicates were performed, and the mean value per experiment was used for statistical analysis. Data are presented as median with quartiles. Asterisks indicate significant differences between LYPS and the other solutions (* *p* < 0.05, ** *p* < 0.01, *** *p* < 0.001, **** *p* < 0.0001, Kruskal–Wallis test).

Mitochondrial dysfunction is an early hallmark of ischemia–reperfusion injury (IRI), often occurring before cell death [16]. To evaluate the protective effect of LYPS on cardiac cells’ integrity, H9C2 cells exposed to control medium at 37 °C were compared to cells exposed to LYPS and standard extracellular preservation solutions (Celsior and Saint Thomas (ST)) at 4 °C during 20 h to assess mitochondrial integrity and cell viability by flow cytometry. We first assessed mitochondrial membrane potential (ΔΨm) with DilC-1 staining in H9C2 cells following an in vitro prolonged cold ischemia–reperfusion sequence (20 h at 4 °C followed by 2 h of reperfusion at 37 °C). Cells preserved in LYPS maintained a proportion of cells with preserved ΔΨm comparable to that observed in control cells (Figure 1A), whereas cells preserved in other extracellular solutions (Celsior or ST) were associated with a significant loss of their mitochondrial membrane potential. These results suggest that LYPS preserves mitochondrial integrity during prolonged cold ischemia–reperfusion. We next assessed whether the maintenance of ΔΨm is associated with improved cell survival assessed by flow cytometry after propidium iodide (PI) staining. Our results showed that LYPS-preserved cells had the lowest percentage of cell death compared to the control (Figure 1B), while cells exposed to Celsior or ST showed significantly higher levels of PI-positive cells. Together, these data indicate that LYPS maintains cell integrity during prolonged cold ischemia–reperfusion by preserving mitochondrial function and reducing cell death.

### 2.2. Effect of LYPS During Prolonged Deep Hypothermic Static Storage and Normothermic Reperfusion on Langendorff Ex Vivo Pig Heart Model

#### 2.2.1. Myocardial Tissue Impairments During Prolonged SCS

▪ Mitochondrial function after 20 h of SCS

**Figure 2:**
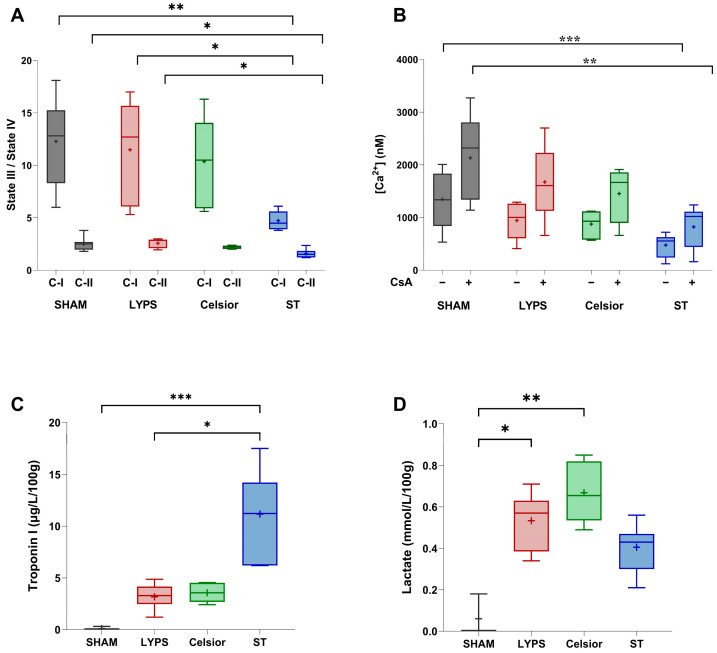
Mitochondrial function and biochemical markers of cardiac injury during SCS preservation. After a 20 h conservation period in the tested solutions, mitochondria were isolated from a ventricular biopsy and the mitochondrial respiratory control index (RCI) was assessed for complex I (C-I) and complex II (II) (**A**). Mitochondrial Ca^2+^ retention capacity (CRC) was assessed with or without the addition of 1 mM cyclosporin A (CsA) (**B**). For the quantification of cardiac tissue damage at the end of SCS, hearts were washed, arrested, harvested, and preserved for 20 h with LYPS, Celsior, or ST. Measurements were performed at the end of cold ischemia in the preservation solution: troponin I release (**C**) and lactate concentration (**D**). Data are presented as median values with quartiles, and the mean is represented by “+”. Sample size: n = 6. Asterisks indicate significant differences between the control, LYPS, and the other solutions: * *p* < 0.05, ** *p* < 0.01, and *** *p* < 0.001 were determined by one-way ANOVA for parametric tests (**A**,**B**) or Kruskal–Wallis for non-parametric tests (**C**,**D**).

Given that mitochondrial dysfunction is an early hallmark of ischemia–reperfusion injury [16], we evaluated mitochondrial function on porcine isolated mitochondria at the end of SCS. Our results showed that cold storage in ST significantly affected complex I and II activity compared to both the LYPS and control groups. Hearts preserved in Celsior were not different from the control, LYPS and ST groups. Surprisingly, mitochondrial respiration measurements obtained with LYPS were very close to the control values, suggesting no loss of function for this parameter after 20 h of deep hypothermia (Figure 2A). These results suggest that LYPS is suitable to preserve mitochondrial respiration during ex vivo prolonged cold ischemia. We next assessed calcium retention capacity (CRC) to evaluate whether mitochondrial Ca^2+^ handling remained intact. No significant difference was observed between the LYPS and control groups, with or without the addition of CsA on mitochondria, whereas the use of ST solution during 20 h of preservation induced a significant decrease in CRC (Figure 2B). Altogether, these results suggest that LYPS solution is suitable to preserve mitochondrial function and Ca^2+^ homeostasis during deep hypothermic preservation.

▪ Biochemical markers of cardiac injury after 20 h of SCS

In order to verify whether preserved mitochondrial function in LYPS was associated with reduced cardiac injury, we measured key biochemical markers of cardiac damage. First, we quantified troponin I release into the perfusate. Our findings indicated a lack of substantial elevation in troponin I release after 20 h of preservation in the LYPS, control, and Celsior groups. However, SCS in ST solution significantly increased troponin I release (Figure 2C). We next assessed perfusate lactate levels and observed that SCS in both LYPS and Celsior groups was associated with increased lactate levels compared to the control group. In contrast, no significant effect was observed in the ST group (Figure 2D). Altogether, these findings suggest that the LYPS solution effectively mitigates myocardial tissue damage and metabolic stress during prolonged SCS.

#### 2.2.2. Functional Recovery After 20 h of SCS and 1 h Ex Vivo Reperfusion

**Figure 3:**
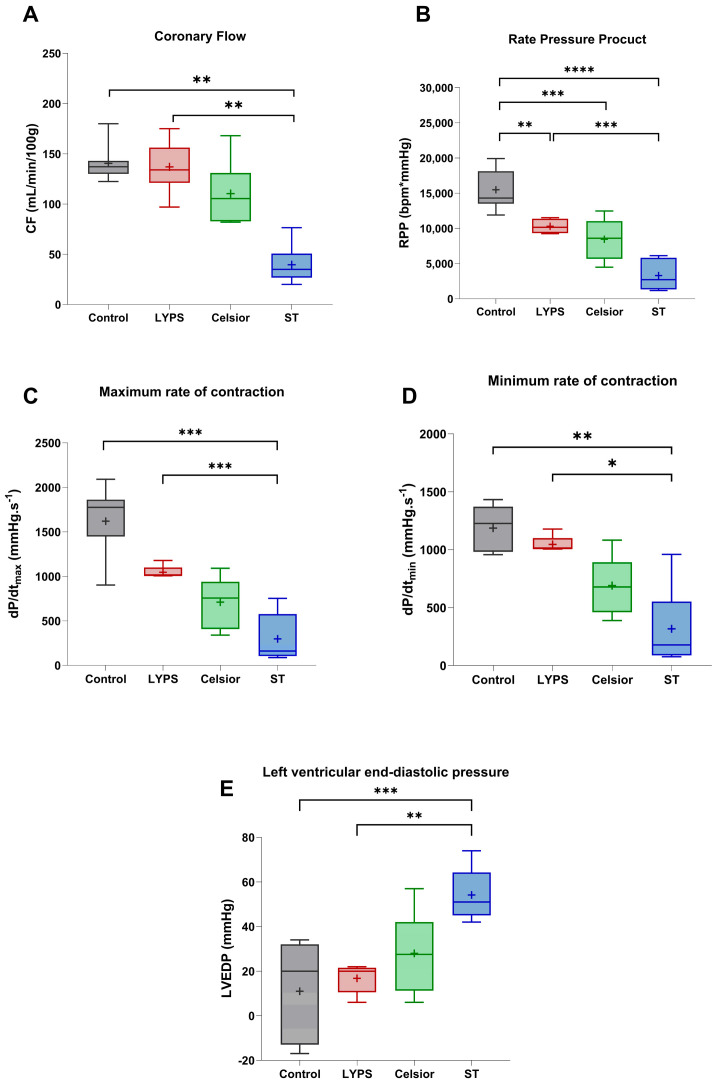
The impact of preservation solutions on the functional recovery of pig hearts after 20 h of CSS. After a 20 h conservation period in the different solutions (LYPS, Celsior, St. Thomas), cardiac functional recovery was assessed by measuring key hemodynamic parameters during the reperfusion period: coronary flow (CF) (**A**), rate pressure product (RPP) (**B**), cardiac contractility (Max dP/dt) (**C**) or relaxation (Min dP/dt) (**D**), and left ventricular end-diastolic pressure (LVEDP) (**E**). Values are shown as median with quartiles, with the mean represented by “+”. Sample size: n = 6. Asterisks indicate significant differences between the control, LYPS, and the other solutions: * *p* < 0.05, ** *p* < 0.01, *** *p* < 0.001, **** *p* < 0.0001 were determined by one-way ANOVA for parametric tests (RPP and diastolic pressure) or Kruskal–Wallis for non-parametric tests.

To assess the overall impact of the LYPS solution on cardiac function after 20 h of SCS and 1 h of reperfusion, we examined key hemodynamic parameters using a Langendorff ex vivo pig heart model (Figure 3A–E). Coronary flow (CF) was measured after 20 h of SCS, and no significant differences were observed between both the LYPS and Celsior groups compared to the control group. However, the ST group showed a significant decrease in CF (Figure 3A). Moreover, the rate pressure product (RPP) was significantly impaired compared to the control group. Hearts preserved in LYPS exhibited better functional recovery than those preserved in ST solution. There was no difference in RPP between hearts preserved in Celsior and those preserved in LYPS (Figure 3A). Furthermore, an analysis of left ventricular pressure dynamics revealed no significant difference in dP/dtmax or dP/dtmin between the LYPS and control groups. In contrast, hearts preserved in ST solution showed a significant decrease in dP/dt_max_ and dP/dt_min_ compared to both the LYPS and control groups (Figure 3C,D). Moreover, hearts preserved in Celsior had dP/dt_max_ and dP/dt_min_ that were reduced by almost 50%, though this difference did not reach significance compared to both the control and LYPS groups. Regarding left ventricular end-diastolic pressure (LVEDP), our results showed a significant rise in the ST group compared to both the control and LYPS. Results for the Celsior group are more mixed and not significantly different to those of the ST group (Figure 3E). Altogether, these results suggest that LYPS solution preserves ventricular function, as dP/dt_max_ and dP/dt_min_ and LVEDP remained unchanged, suggesting that LYPS maintained systolic and diastolic function and normal ventricular compliance.

#### 2.2.3. Effects on Rhythmic Function

**Figure 4:**
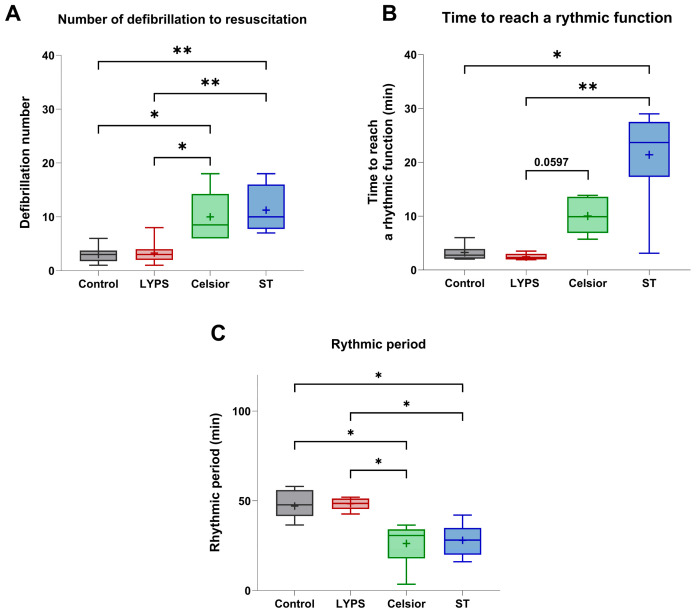
The impact of preservation solutions on the rhythmic function of pig hearts after 20 h of CSS. After a 20 h conservation period in the different tested solutions (LYPS, Celsior, or ST), ex vivo reperfusion was realized at 37 °C for 1 h using the Langendorff system. Rhythmic parameters were analyzed during the reperfusion: number of defibrillations (**A**), time to reach a rhythmic function (**B**), and rhythmic period (**C**). Values are shown as the median with quartiles, with the mean represented by “+”. Sample size: n = 6. Asterisks indicate significant differences between the control, LYPS, and the other solutions: * *p* < 0.05, ** *p* < 0.01, determined by one-way ANOVA for parametric tests (the number of defibrillations) or Kruskal–Wallis for non-parametric tests (time to reach a rhythmic function and rhythmic period).

To assess the impact of LYPS solution on rhythmic function, we next evaluated several rhythmicity parameters. Hearts preserved in LYPS exhibited no alteration in defibrillation number and time to reach a rhythmic function compared to the control group (Figure 4A–C). Conversely, hearts preserved in Celsior or ST showed a substantial increase in the number of defibrillations required to restore rhythmic activity compared to the control and LYPS groups (Figure 4A). Similarly, the time to reach a stable rhythmic function was significantly prolonged in the ST group relative to both the LYPS and control groups (Figure 4B). Interestingly, the rhythmic period was significantly reduced in the Celsior and ST groups compared to the LYPS and control groups (Figure 4C). Our findings indicate that LYPS solution is associated with improved cardiac rhythmic function, as it does not adversely affect rhythm recovery during ex vivo reperfusion after prolonged deep hypothermic static storage.

#### 2.2.4. Assessment of the Electrophysiological Properties of Adult Pig Cardiomyocytes

**Figure 5:**
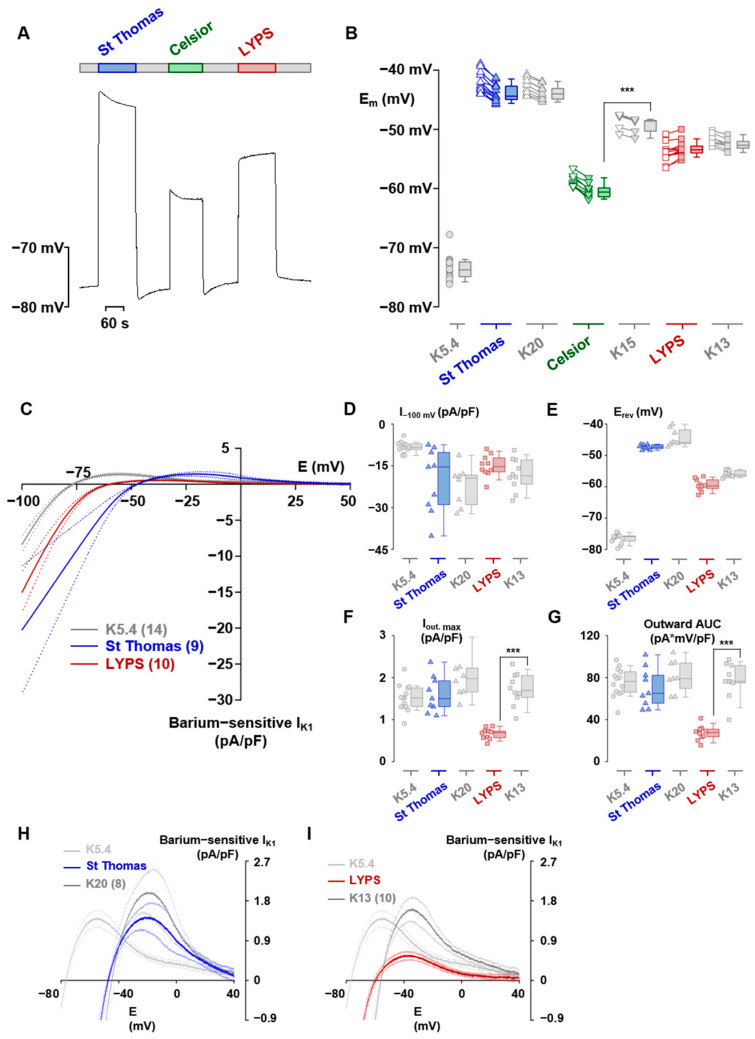
Electrophysiological effects of LYPS, ST and Celsior on pig adult rat cardiomyocytes. Representative effects of ST, Celsior and LYPS preservation solutions on cardiomyocyte membrane potential (**A**). Membrane potentials of cardiomyocytes measured in preservation solutions as in A and in equivalent potassium media (**B**). Empty symbols correspond to membrane potentials measured just after the change in solution. Solid symbols correspond to measurements taken at the end of expired medium. Data are presented as the median with quartiles. N = 18 for the K5.4 solution, 12, 9 and 12 for the ST, Celsior and LYPS and 9, 5 and 8 for their equivalent potassium media. Superimposed mean current–voltage relations of the barium-sensitive IK1 established from descending ramps in K5.4 solution, ST and LYPS. Data are expressed as mean ± standard deviation (**C**). (**D**–**G**) I_K1_ current amplitude at −100 mV (I−100 mV, panel (**D**)), reversal potential (E_rev_, panel (**E**)), peak outward current (I_out_, max, panel (**F**)) and area under the outward part of the curve (Outward AUC, panel (**G**)) established from the same cells as in C for ST and LYPS solutions and for their equivalent potassium media. Data are presented as the median with quartiles. (**H**,**I**) The magnified outward part of I_K1_ for ST (panel (**H**)) and LYPS (panel (**I**)) solutions and for their equivalent potassium media. *** *p* < 0.001 determined by a non-parametric Kruskal–Wallis test.

To explore the anti-arrhythmic properties of LYPS solution, we conducted electrophysiological assessments on isolated pig cardiomyocytes (Figure 5). We first measured the effect of preservation solutions on the membrane potential of isolated cardiomyocytes. We observed that the presence of LYPS or ST in the cells resulted in immediate depolarization consistent with their respective potassium concentrations. Conversely, depolarization induced by Celsior was of lesser amplitude than expected (Figure 5A,B). Additionally, we observed progressive repolarization (by ~2 mV) in ST, Celsior and all three equivalent potassium media, whereas slow depolarization (by ~1 mV) occurred in LYPS. Each of these slow transients appeared in the reverse direction after returning to the control medium (Figure 5A). With respect to the significant impact of the background inward rectifier current (I_K1_) on the membrane potential, we recorded the full current–potential relationship of the barium-sensitive current under control, ST, and LYPS conditions (Figure 5C). We measured the I_K1_ amplitude at −100 mV (I_−100 mV_), its reversal potential (E_rev_), the peak of the outward current (I_out_, _max_) and the area under the outward part of the curve (Outward AUC). This was conducted for the control, ST, LYPS, and their equivalent potassium media (Figure 5D–G). Only in the case of LYPS, and only regarding I_out_, _max_ and Outward AUC (Figure 5F,G), was there a significant difference between a solution and its equivalent potassium medium. Thus, only LYPS caused a sizeable diminution of the outward I_K1_ (Figure 5H,I). These findings indicate that LYPS specifically modulates the outward component of the I_K1_ current in cardiomyocytes, which may contribute to its anti-arrhythmic properties by subtly slowing repolarization.

## 3. Discussion

Heart transplantation remains the gold standard therapy for end-stage heart failure [1], yet its success is limited by the susceptibility of donor hearts to ischemia–reperfusion injury during cold storage [17]. Extending safe preservation time is critical for improving graft availability, enabling long-distance allocation, and reducing primary graft dysfunction [18,19]. Over the past few decades, several preservation solutions—including St. Thomas, University of Wisconsin (UW), Custodiol (HTK), and Celsior—have been developed to mitigate ischemic injury, but none have fully eliminated functional deterioration during prolonged storage. In this context, we investigated the efficacy of LYPS, developed in our laboratory, as a novel preservation strategy for long-term preservation with static cold storage. In our study, we demonstrated that LYPS is able to provide good myocardial protection during prolonged SCS (20 h at 4 °C) followed by reperfusion. Using complementary models including immortalized cardiomyoblasts (H9C2), isolated porcine hearts, cardiac mitochondria and primary cardiomyocytes, we demonstrated that hearts preserved in LYPS exhibited greater cell viability and functional recovery, enhanced contractility and more stable rhythmicity, with almost no need for defibrillation shocks. These findings suggest that LYPS effectively preserves myocardial viability and function, highlighting its potential to improve cardiac graft quality during extended SCS.

At the cellular level, our results demonstrate that LYPS provides effective protection during prolonged hypothermic simulated conservation. In H9C2 cells, viability was better maintained, as indicated by reduced cell death and preserved mitochondrial membrane potential compared to the control. On isolated mitochondria isolated from pig hearts at the end of reperfusion, LYPS also supported the preservation of mitochondrial function, as shown by the sustained activity of complexes I and II, as well as the maintenance of calcium retention capacity (CRC), similar to the control and Celsior groups. This indicates that LYPS is able to maintain mitochondrial functions for up to 20 h of prolonged SCS, highlighting its efficacy under conditions where cellular stress is exacerbated. LYPS was designed to increase glycolytic pathways. Indeed, LYPS contains metabolic substrates and metabolically active hormones (such as glucose, pyruvate, glutamate, and insulin) that can directly activate the glycolytic pathway and mitochondrial functions [20,21,22]. Thus, glycolytic key enzymes, such as PFK and AMPK, can be activated by insulin [23,24]. The Krebs cycle and subsequently the mitochondrial respiratory chain can also be activated by the presence of pyruvate, glutamate and aspartate. Our results confirmed better mitochondrial respiratory index maintenance and the greater resistance of mitochondrial permeability transition pore (mPTP) to opening in the LYPS group, highlighting the maintenance of mitochondrial functions. The analysis of mitochondrial respiration revealed a surprising fact: the mitochondria of hearts kept for 20 h in LYPS are almost identical to the control. There was no loss of function with LYPS at the level of complexes I and II of the respiratory chain. Moreover, the mitochondrial membrane potential, was maintained in cells preserved with LYPS identically to the controls, while it sharply decreased with the other solutions. It is often assumed that SCS markedly suppresses metabolic activity; however, this is not the case. We believe that the presence of metabolic substrates (glucose, insulin, and pyruvate) in LYPS likely supports cellular metabolic activity to be maintained even at 4 °C. Pulis et al. demonstrated that, even under hypothermia and hypoxia, cardiac energy metabolism remains active, with significant glycolytic flux and ATP turnover persisting during SCS [18]. Thus, we hypothesize that certain components of LYPS may confer mitochondrial protection and counteract cell death pathways. In particular, studies have demonstrated that PEG, a constituent of LYPS, reduces apoptosis by stabilizing mitochondrial membranes, thereby preventing the cytoplasmic release of cytochrome c and subsequently limiting the activation of caspases 9 and 3 [19]. PEG is also a macromolecule able to limit extracellular edema. Other substrates in LYPS may confer cardiac cytoprotection. The 40 mM HEPES concentration helps maintain the pH of the solution. Finally, procaine, an antiarrhythmic agent that maintains cardiac arrest, has also been shown to have antioxidant properties.

The apparent maintenance of the ionic homeostasis of cardiomyocytes may result from the convergence of several phenomena. The passive influx of Na^+^ and Ca^2+^ through leakage currents is lowered by the diminution of their conductance at a low temperature and by a lower transmembrane driving force due to depolarization by low K^+^. Maintained ATP production through energized glucose metabolism (metabolic substrates and elevated glucose) will support the activity of the sodium–potassium pump, thus preventing excess intracellular Na+ gain and K^+^ loss, while the inconvenience of lactate production will be buffered by 40 mM HEPES. As a result, the Na^+^ gradient, although diminished, in combination with the limited depolarization (only 13 mM external K^+^), and the low external Ca^2+^ (1 mM) may enable the Na-Ca exchanger to remain in the direct Ca^2+^ extrusion mode [25].

Ex vivo perfusion data further support these findings. Indeed, troponin I release remained unchanged in hearts preserved with both LYPS and Celsior, while its level significantly increased in ST, consistent with the lower functional mitochondrial preservation observed with this solution. Interestingly, lactate levels increased in hearts preserved with both LYPS and Celsior but remained unchanged in ST. Although lactate accumulation is often considered as a marker of metabolic stress [26], in our study—where CRC and mitochondrial function are preserved and troponin I remains unchanged—this increase may reflect preserved metabolic activity rather than cellular injury. This suggests that the heart remains functionally active despite prolonged SCS. The protection of cardiomyocytes from oxidative stress by scavenging free radical species has been reported for lactate in previous studies [27,28,29]. Our findings indicate that preservation in LYPS is associated with improved mitochondrial function, reduced cell death, and limited Ca^2+^ overload. These results are consistent with previous studies highlighting the role of ischemic necrosis and mitochondrial dysfunction as key drivers of graft viability and post-transplant recovery. Esposito et al. showed that the presence of ischemic necrosis in early biopsies after heart transplantation is strongly correlated with impaired graft function and that its severity depends on the preservation solution, being higher with ST than Celsior [9,30].

The present study was conducted using a porcine heart model, chosen for its close anatomical, physiological, and metabolic similarities to the human myocardium. As noted by Jia et al., pigs constitute an optimal large animal model for cardiovascular research, allowing a clinically relevant evaluation of ischemia–reperfusion injury and myocardial preservation [31]. Likewise, Rahman et al. emphasized that large animal models provide a critical translational bridge between small animal studies and clinical practice [32]. Notably, pigs have very limited coronary collateral circulation, closely mirroring human coronary anatomy and permitting reproducible, homogeneous ischemic injury upon perfusion interruption [33,34,35]. This contrasts with rodents, whose microvascular architecture differs substantially, limiting their relevance for modeling cardiac graft preservation. Additionally, the porcine myocardium exhibits a metabolic profile similar to that of humans, relying predominantly on non-esterified fatty acids for energy production [36]. Collectively, these characteristics make the porcine heart an ideal model for testing preservation and reperfusion strategies under clinically relevant conditions.

The initial reperfusion period following the preservation period is crucial for the functional recovery of the graft. Indeed, primary graft dysfunction is the leading cause of early mortality after heart transplantation [18,37]. In the present study, hearts preserved for 20 h in LYPS showed improved ex vivo functional recovery, with higher CF, RPP, ±dP/dt and LVEDP, compared to hearts preserved in ST solution where all these parameters were significantly worse. Interestingly, during reperfusion, coronary flow in the LYPS heart group remained very close to that of the control group, suggesting a high level of vasomotion regulation. We also explored the electrophysiological effect of LYPS on porcine cardiomyocytes. Three notable effects were observed: (i) the slow repolarization seen under both ST and Celsior are likely related to their potassium content, (ii) the slow depolarization seen under LYPS is remarkably opposite to the slow repolarization under its equivalent potassium medium and (iii) only LYPS caused more than a 60% decrease in the outward part of I_K1_. While observations (i) and (ii) deserve further investigation, an exploration of the latter effect might provide useful data to understand the lower incidence of fibrillation upon reperfusion after LYPS preservation. Unlike Celsior and ST, the LYPS solution resulted in a significant 60% reduction in the outward part of the current–voltage relationship of the I_K1_ current. If this effect occurs during reperfusion after cold storage with LYPS, a lengthening of the duration of the ventricular action potential and of the relative refractory period are expected. This change would likely reduce the incidence of re-entry-related fibrillation. Moreover, hearts preserved in LYPS showed sustained rhythmicity and required fewer defibrillations compared to the Celsior or ST groups, highlighting improved electrophysiological stability. At the cellular level, cardiomyocytes preserved in LYPS showed a significant decrease in outward K^+^ current (I_K1_), which would likely contribute to prolonged action potential and improved Ca^2+^ retention in the cytosol, supporting contractile function and rhythmic stability [38]. Moreover, ATP production and Ca^2+^ handling are critical for maintaining excitation–contraction coupling, and disturbances in these processes are known to contribute to arrhythmias and contractile dysfunction following ischemia–reperfusion [39,40,41].

Overall, these data suggest that improving preservation solutions to support mitochondrial function and ionic homeostasis is important for reducing ischemic necrosis and allowing a better recovery after reperfusion. These findings align with previous reports comparing extracellular and intracellular solutions. A previous study of our group showed that the SCS preservation of rat hearts for 8 h in extracellular solutions (Celsior, ST, etc.) was more effective than in intracellular solutions (UW, Custodiol) [13]. However, cardioplegic solutions like ST are less suitable for prolonged preservation as they are associated with lower one-year survival and higher rejection rates compared to Celsior [9].

The 20 h hypothermic preservation used in our study represents a very long extended storage time compared to clinical practice, where cardiac grafts are usually stored for 4 to 6 h [42]. Prolonged hypothermia, while reducing metabolic rates, does not completely suppress metabolism [43,44,45], leading to substrate depletion. Thus, SCS beyond 6 h has been associated with severe energetic depletion, as shown in human hearts preserved for 12 h [46], and metabolic stress increases with longer storage [44]. Therefore, prolonged hypothermic storage is generally considered to impair myocardial homeostasis and increase susceptibility to reperfusion injury. Surprisingly, the LYPS solution (and, to a lesser extent, the Celsior solution) demonstrated its ability to maintain ionic homeostasis, mitochondrial metabolism and cell integrity over a very long period of SCS, whereas cardioplegic solutions such as ST failed to sustain cellular homeostasis, leading to more severe graft injury. In this context, our solution appears particularly effective in preserving heart graft viability. Overall, these findings highlight the ability of the LYPS solution to maintain cellular hemostasis during extended hypothermic storage.

### Limitations of the Study

Our study has some limitations. First, the experiments were performed using ex vivo and in vitro models, which do not reproduce the inflammatory response and neuro-hormonal responses of in vivo transplantation. In fact, hearts were not subjected to brain death prior to retrieval, which may influence metabolism, oxidative stress, and endothelial activation. Second, our analysis was limited to short-term reperfusion (1 h); therefore, long-term performance remains to be evaluated.

The retrograde Langendorff-perfused heart remains a key tool in cardiovascular research, offering precise control of hemodynamics and metabolism while isolating the heart from systemic influences [47,48]. It is particularly valuable for studying cardiac grafts subjected to ischemia–reperfusion or prolonged hypothermic/subnormothermic preservation, as it allows for an assessment of viability, contractile function, and coronary flow without imposing the high metabolic demands of physiological preload and afterload, enabling a distinction between primary preservation injury and secondary dysfunction [49]. In contrast, the working heart model reproduces physiological loading and enables an integrated evaluation of mechanical performance, but it has intrinsic limitations: it requires a fully oxygenated, energetically competent myocardium, is highly dependent on operator skill, is very sensitive to perfusion and temperature variations, and shows lower reproducibility and higher variability [49]. These limitations, taken together with the additional metabolic load, limit its suitability for marginal or ischemia-challenged grafts. Overall, while the working heart provides the most physiologically relevant assessment in viable hearts, the Langendorff system remains the method of choice for graft preservation studies. It allows a reliable investigation of intrinsic myocardial recovery, energy metabolism, and molecular signaling under conditions where physiological loading could mask or exacerbate preservation-induced alterations. Nevertheless, further validations in working heart models are needed, as differences in metabolic and mechanical demands may influence ischemia–reperfusion injury, cardiac metabolism, Ca^2+^ homeostasis, and arrhythmia susceptibility.

## 4. Materials and Methods

### 4.1. In Vitro Experiments

#### 4.1.1. H9C2 Cell Culture

In vitro experiments were conducted on two cell models: H9C2-SV40 rat embryonic cardiomyoblasts (purchased from ATCC, Molsheim, France: ATCC^®^ CRL-1446™ and immortalized with SV40) as previously described [50] and isolated adult pig cardiomyocytes. H9C2 cells were cultured in complete DMEM (Dulbecco’s Modified Eagle Medium, Gibco-Thermofisher Scientific, Bourgoin, France) supplemented with 10% fetal bovine serum and 1% antibiotic mixture (penicillin/streptomycin, Gibco Thermofisher Scientific, France). Culture media were renewed every two days. Cells were maintained in a 37 °C incubator under a controlled atmosphere containing 5% CO_2_.

#### 4.1.2. Preservation Solutions

The composition of all preservation solutions used for our experiments is showed in Table S1.

#### 4.1.3. In Vitro Simulated Ischemia–Reperfusion Injury Protocol

In order to mimic the preservation–reperfusion sequence undergone by cardiac grafts prior to transplantation, cells were stored in various preservation solutions (Figure S1). Day 1: cell seeding. H9C2-SV40 were seeded at 27,000 cells/cm^2^ in complete medium and placed in the incubator at 37 °C with 5% of CO_2_. Day 2: preservation period. The culture medium was removed, and after 3 washes with PBS to remove all traces of nutrients, the cells were incubated in control medium (NaCl), LYPS solution, or standard extracellular solutions (Celsior and St. Thomas) and stored at 4 °C for 20 h. This preservation sequence was used to mimic the cold storage period present during graft preservation before transplantation. Day 3: reperfusion period. After the preservation period, complete culture medium was added on the cells for 2 h to simulate reperfusion when the organ was transplanted.

#### 4.1.4. Mitochondrial Membrane Potential (ΔΨm)

Mitochondrial membrane potential was assessed using 2 nM 1,1′,3,3,3′,3′-hexamethylindodicarbo-cyanine iodide (DilC1) (ex 638 nm/em 658 nm) (Enzo Life science, Lyon, France). Briefly, cells were collected and washed twice with PBS. Cells were then incubated with 2 nM DILC1 for 30 min at 37 °C. After staining, cells were washed and resuspended in PBS and immediately analyzed by flow cytometry. Mitochondrial depolarization was evaluated based on the fluorescence intensity of DilC1.

#### 4.1.5. Propidium Iodide (PI) Staining for Cell Death

Cell necrosis was evaluated with 1 μg/mL propidium iodide (PI) (Sigma-Aldrich, Saint-Quentin-Fallavier, France), (ex 535 nm/em 620 nm) as previously described [51,52].

#### 4.1.6. Flow Cytometry Analysis

After the preservation–reperfusion sequence, flow cytometry experiments were performed to assess cell death and mitochondrial membrane potential. Data were obtained using BD Fortessa X-20, with at least 10,000 events recorded per sample. Data acquisition and analysis were performed using Flow-jow 10.8.0 Software (BD Biosciences, Le Pont de Claix, France), and appropriate gates were applied to exclude debris and doublets. Experiments were performed in triplicate.

### 4.2. Ex Vivo Experiments (Pig Heart Preservation–Reperfusion Sequence)

#### 4.2.1. Animals

All experiments were performed in accordance with the recommendations of the European Ethical Committee (EEC) (2010/63/EU), the French National Ethical Committee (87/848), the National Society for Medical Research and the ‘Guide for the Care and Use of Laboratory Animals’ prepared by the Institute of Laboratory Animal Resources and published by the National Institutes of Health (NIH Publication No. 86-23, revised 1996). Protocols were approved by the Lyon 1 University animal care committee under references APAFIS#16758_201809171143620. 

Twenty-four farm pigs of both sexes weighing 35 ± 5 kg were randomly allocated to one of the following groups (n = 6 per group): control, LYPS, Celsior, or St. Thomas.

##### Surgical Procedure

Anesthesia was induced via an intravenous injection of a ketamine–xylazine mixture (8 mL total; 4 mL ketamine, 50 mg/mL and 4 mL xylazine, 10 mg/mL). Supplemental anesthesia was administered using a bolus of propofol (1%, 6 mL) and fentanyl (10 µg/mL, 1 mL). Following induction, all animals were intubated and mechanically ventilated with a mixture of oxygen and air, supplemented with 3.5% sevoflurane. Median sternotomy was performed, and a catheter was inserted into the ascending aorta. Heparin (1000 IU/kg) was administered intravenously, followed by cardiac arrest induced via an intra-aortic injection of 1 L of cold (4 °C) preservative solution (LYPS, Celsior, or St. Thomas). The compositions of these solutions are listed in Table S1. Hearts were subsequently excised and stored in 1 L of the same solution at 4 °C for 20 h. In the control group, hearts were harvested and immediately reperfused after a brief preservation period of less than 1 h at 4 °C (Figure S2).

##### Ex Vivo Simulated Ischemia–Reperfusion Injury Protocol

After flushing, all hearts were immersed in 1.L of preservation solution and subjected to 20 h of static cold ischemia (4 °C). Following this ischemia period, hearts underwent reperfusion for 1 h at 37 °C, except for the control group, which was reperfused less than 1 h after harvesting.

#### 4.2.2. Assessment of Cardiac Tissue Damage

##### Markers of Cardiac Distress

Markers of cardiac distress and mitochondrial function were assessed at the end of the cold storage period. Cellular injury was evaluated by measuring the release of lactate (mM) and troponin I (IU/mL/mg tissue) in the conservation solution at the end of the cold ischemic period [53,54,55,56]. Biochemical analyses were performed at the Biology and Biochemistry Department of Louis Pradel Hospital.

##### Mitochondrial Function Assessment

Mitochondrial oxygen consumption rate: At the end of cold storage, ventricular biopsies were collected, and mitochondria were isolated as previously described [57]. The oxygen consumption of freshly isolated mitochondria was measured at 25 °C with a Clark electrode (Oroboros Oxygraph, Innsbruck, Austria). Mitochondria (250 µg proteins) were incubated in respiratory buffer (50 mM Tris/HCl, 100 mM KCl, 5 mM KH2PO4, 1 mM EGTA) supplemented with 0.1% BSA, pyruvate, malate and glutamate (5 mmol/L each) as substrates to provide electrons to complex I or succinate for complex II. State III (200 µmol/L ADP addition) and state IV (ADP limited) were assessed and the respiratory control index (RCI = State III/State IV) was determined, as previously described [52].

Calcium retention capacity (CRC): CRC was determined fluorometrically using 0.25 µM Calcium Green-5N (Life technology, Thermofisher scientific, France). Isolated cardiac mitochondria were suspended in CRC buffer containing 150 mM sucrose, 50 mM KCl, 2 mM KH2PO4, 20 mM Tris/HCl, 5 mM succinate–Tris. Sequential CaCl_2_ pulses (10 µM) were applied until a rapid and sustained fluorescence increase indicated mitochondrial permeability transition pore (mPTP) opening. CRC was expressed as the cumulative Ca^2+^ load (nmol·mg^−1^ protein) required to trigger a massive Ca^2+^ release by mPTP. Cyclosporin A (1.25 µM) was used in parallel assays to confirm mPTP dependence.

#### 4.2.3. Assessment of Functional Recovery

After 20 h of static cold storage, a latex balloon was introduced into the left ventricle and connected to a pressure transducer with a recorder (Power Lab - AD instruments, Oxford, UK). Hearts were then reperfused retrogradely by the aorta on a Langendorff system with 600 mL conserved, heparinized whole blood at 38 °C from the same pig. The initial pH of the blood was 7.2 to 7.4 after equilibrium with a mixture of 95% O_2_/5% CO_2_ and 20 mL Na_2_CO_3_ at 4.2%. Perfusion pressure was gradually increased from 30 cm H_2_O to 100 cm H_2_O along the first 10 min of reperfusion and then maintained until the end of reperfusion time (60 min). At baseline and every 10 min during reperfusion, the functional state of the heart was evaluated by measuring the heart rate (HR), left ventricular systolic pressure (LVSP) and left ventricular end-diastolic pressure (LVEDP). The rate pressure product [RPP = (LVSP − LVEDP) × HR], maximum rate of rise in the LV pressure (dP/dt max) and maximum isovolumetric rate of relaxation (dP/dtmin) were calculated. Coronary flow (CF) was measured by collecting the coronary effluent every ten minutes.

#### 4.2.4. Assessment of Rhythmic Parameters

Pig ventricular myocytes were isolated by excising a segment of the left ventricular wall and perfusing it through its cannulated feeding artery under constant pressure. Perfusion was performed as follows: first, for 10 min with oxygenated, pre-warmed (37 °C) low-Ca^2+^ Tyrode solution (solution A) containing 140 mM NaCl, 4 mM KCl, 0.03 mM CaCl_2_, 2 mM MgCl_2_, 0.4 mM NaH_2_PO_4_, 5 mM Na-pyruvate, 10 mM glucose, and 10 mM HEPES, pH adjusted to 7.4 with NaOH; second, for 40–60 min with solution A supplemented with 280 U/mL collagenase (Worthington-Thermofisher Scientific, France) and 90 U/mL hyaluronidase (Sigma-Aldrich, Saint-Quentin-Fallavier, France); third, for 5 min with solution A containing 1.2% bovine se-rum albumin (Sigma); and finally, for at least 10 min with KB medium [58]. The digested tissue was cut into thin lamellae and gently stirred in KB medium for 15–20 min to release individual myocytes. Isolated cells were filtered through a 200 µm mesh and stored at 4 °C in KB medium for at least 2 h before use.

#### 4.2.5. Electrophysiology

Membrane potentials and currents were recorded at room temperature (20–23 °C) with the conventional (ruptured patch) whole-cell patch clamp technique using the Axopatch 200B amplifier and pClamp 11 software via the Digidata 1550B acquisition system (Molecular Devices, Workingham, UK). Cardiac cells were placed on the stage of an inverted microscope in a plastic Petri dish containing solution A whose Ca^2+^ concentration was increased to 1 mM. A set of six capillaries connected to a single outlet was placed close to the cell to be recorded to continuously perfuse it with gravity-flowing solutions (flow rate of 20–40 µL/s) and allow a rapid change (within 5 s) in the external medium. Cells were allowed to adhere for several minutes before perfusion was initiated with the following external solution: K5.4 medium, 140 mM NaCl, 5.4 mM KCl, 2 mM CaCl_2_, 2 mM MgCl_2_, and 10 mM glucose, 10 mM Hepes, adjusted to pH 7.4 with NaOH. Patch pipettes, with resistances of 1–4 MΩ when filled with the following internal medium, 135 mM KCl, 3 mM MgATP, 5 mM EGTA, 5 mM glucose, and 10 mM Hepes, adjusted to pH 7.2 with KOH, were used routinely. Membrane capacitance (Cm) was systematically measured and was calculated by analyzing the capacitive surge produced by a small voltage step as previously described [59]. The background inward rectifier current (I_K1_) was recorded as the barium-sensitive current using the following voltage clamp ramp-based protocol. From a holding potential of −80 mV, a 520 ms depolarizing voltage ramp to +50 mV (0.25 V/s) was followed by a 600 ms reverse ramp to −100 mV (−0.25 V/s) and then by an 80 ms depolarizing ramp to return to −80 mV. Membrane currents were evoked every 10 s, sampled at 10 kHz and low pass-filtered at 3 kHz. Current traces were uncorrected for the leak. Current–voltage relationships of I_K1_ normalized to Cm were constructed from data acquired during the repolarizing voltage ramp and it was verified on several cells that these curves were like those established using a conventional rectangular pulse voltage protocol (Figure S3A–C). As indicated, this type of ramp-based protocol could be repeated frequently without damaging the patched cell, enabling the effects of different cardioplegic solutions on the I-V curve of I_K1_ to be tested [60]. To determine whether two groups of data were statistically different, an unpaired t-test was conducted using GraphPad Prism 10.6.0 software. A difference was assumed to be statistically significant if *p* < 0.05.

### 4.3. Statistical Analysis

Data were expressed as mean ± standard deviation (SD) or the median with interquartile range (IQR), depending on the distribution of the data. The normality of the data was assessed using the Shapiro–Wilk test. Differences between groups were evaluated using one-way ANOVA and the Kruskal–Wallis test, as appropriate for the type of data and the study design. For a comparison of more than two groups, one-way analysis of variance (ANOVA) followed by Tukey’s post hoc test was performed for parametric data. When data did not meet the assumptions of normality, the non-parametric Kruskal–Wallis test was used, followed by Dunn’s post hoc test. Statistical significance was considered when *p*-values were less than 0.05. All statistical analyses were performed using GraphPad Prism.

## 5. Conclusions

In summary, the present study demonstrates that LYPS confers a significant protective effect against prolonged hypothermic preservation–reperfusion injury across both cellular and ex vivo pig models by limiting cell death, restoring a stable cardiac rhythm—possibly through mechanisms involving I_k1_ current modulation—and enabling the successful extension of SCS up to 20 h without a machine perfusion. LYPS emerges as a promising strategy to improve graft preservation. These findings highlight its potential translational relevance for heart transplantation, where extending safe preservation times remains a major clinical challenge.

## Data Availability

The original contributions presented in this study are included in the article/Supplementary Material. Further inquiries can be directed to the corresponding author.

## Supplementary Materials

The supporting information can be downloaded at https://www.mdpi.com/article/10.3390/ijms262211170/s1.

## Abbreviations

The following abbreviations are used in this manuscript:

LYPS	Lyon preservation solution
ST	St. Thomas
CSS	Cold static storage
Δψm	Mitochondrial membrane potential
mPTP	Mitochondrial permeability transition pore
