# Supplementary material for: Extended Preservation of Heart Grafts: LYPS Solution Maintains Cardiac Function During 20-Hour Static Cold Storage
Source: Int J Mol Sci. 2025 Nov 19;26(22):11170. doi: 10.3390/ijms262211170 (PMC12652780; doi:10.3390/ijms262211170)
Supplement: Supplementary file 1 [file ijms-26-11170-s001.zip › ijms-3933565-supplementary.pdf]

SUPPLEMENTAL DATA

Category	Components	LYPS*	Celsior	St Thomas (ST)
**Electrolytes** (mM)	Na^+	120	100	144
	K^+	13	15	20
	Ca^{2+}	1	0.25	2.4
	Mg^{2+}	4	13	16
	Cl^-	130	30	203
**Impermeants** (mM)	Mannitol		60	
	Lactobionate		80	
	Raffinose			
	HES			
	PEG8000	0,25		
	PEG20000	0,15		
**Buffer** (mM)	Histidine		30	
	Phosphate			
	Bicarbonate			10
	HEPES	40		
**Antioxidants** (mM)	Glutathione		3	
**Metabolic substrates** (mM)	α-ketoglutarate	1.4		
	Glutamate		20	
	Adenosine			
	Glucose	20		
	Aspartate	2		
	Pyruvate	2.5		
	Insulin	250 UI/L		
	Tryptophan			
**Other components** (mM)	Potassium hydroxyde	1		
	Procaïne hydrochloride	1		1
**Parameters**	pH	7.35	7.3	7.8
	Osmolarity	320	320/360	290

Table S1: Composition of the various preservative solutions used is presented. It is important to note that the LYPS solution used in this study is a new formulation, which differs from those in previous publications (1,2).

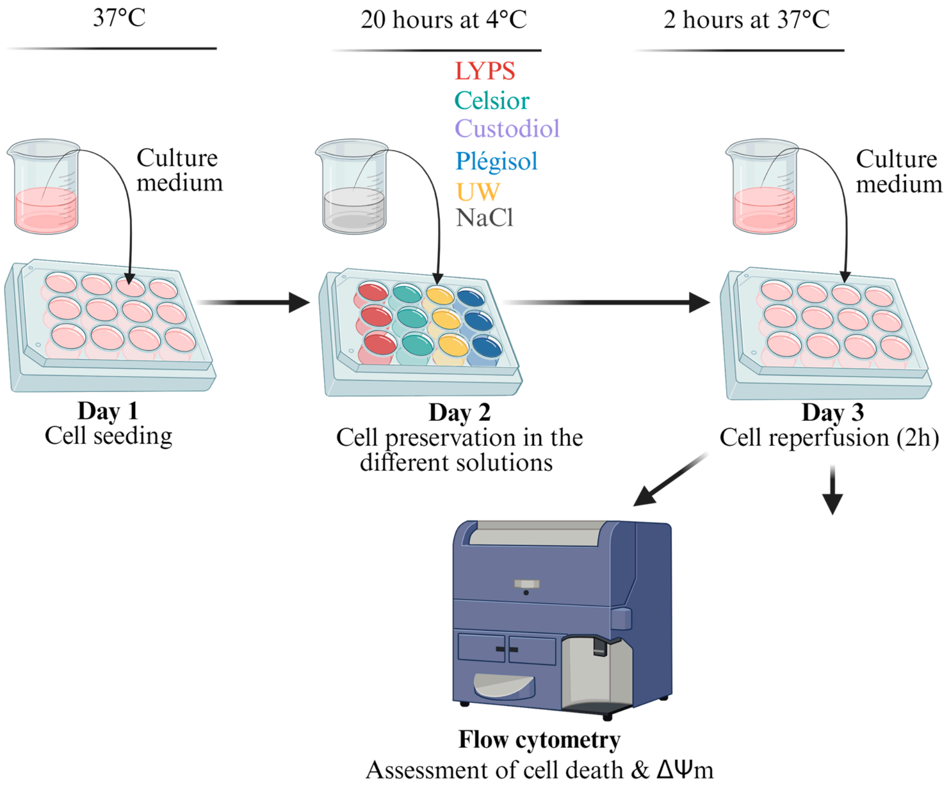

Figure S1: Cardiac cells preservation-reperfusion sequence (Created with BioRender.com):

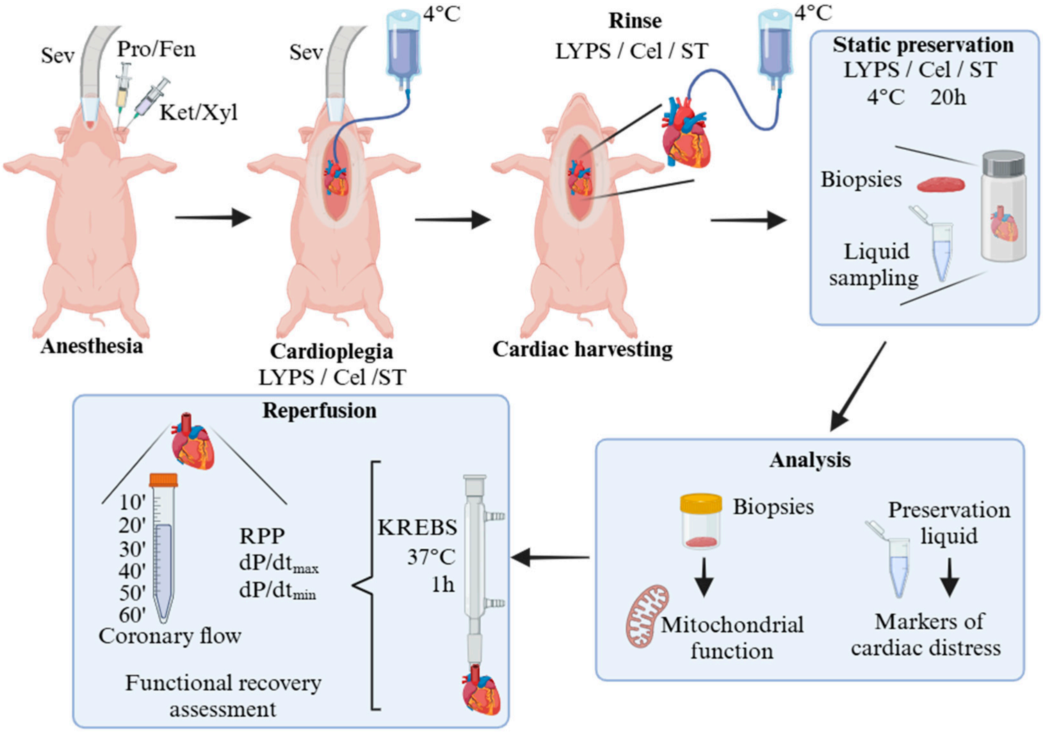

Figure S2: Methods for ex-vivo pig hearts preservation-reperfusion sequence. (Sev: sevoflurane, Pro: propofol, Fen: Fentadon, Ket: ketamine, Xyl: xylazine, Cel: Celsior, ST: St Thomas) **(**Created with BioRender.com).

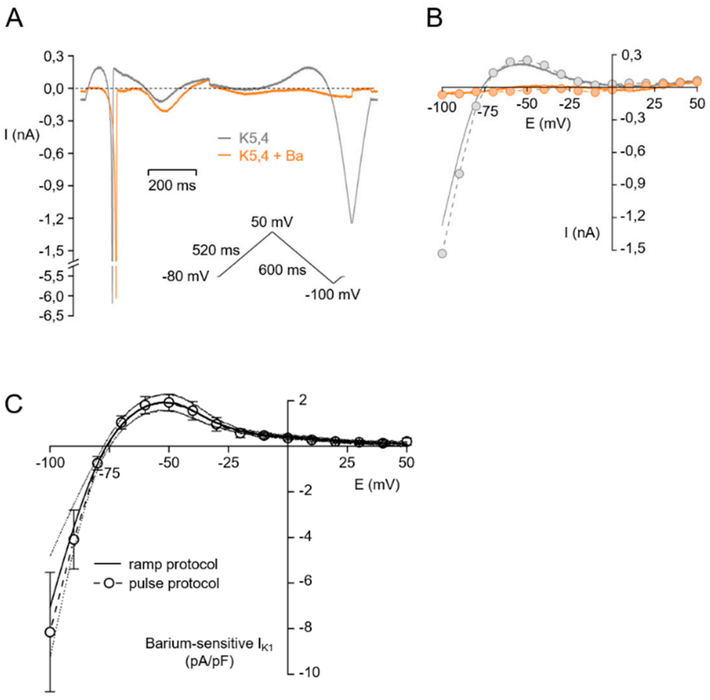

Figure S3: Electrophysiology on freshly isolated pig ventricular cardiomyocytes

Current responses to voltage-clamp upgoing and downgoing ramps, as shown in the inset, recorded in control conditions (black line) and in the presence of 4 mM barium (red line). During the upgoing ramp, the rapid inward Na^+ current and the slower Ca^{2+} current are sequentially activated and then inactivated. As a result, during the downgoing ramp, only outward K^+ currents together with leakage and background currents remain (**A**). The current responses to the downgoing ramp from panel A are plotted as a function of the corresponding ramp voltages. The dots connected by dashed lines represent the currents recorded in the same cell at the end of 500-ms square pulses delivered from a holding potential of –40 mV, a condition used to inactivate inward Na^+ currents. Black dots correspond to control values, and red dots to values obtained in the presence of 4 mM barium (**B**). Barium-sensitive I_{K1} currents extracted from ramp protocols (solid line, with 95% confidence intervals shown as dotted lines) and from pulse protocols (dots, with 95% confidence intervals shown as vertical bars). Data represent the average values obtained from the same eight cells for both sets of measurements (**C**).

Supplemental References:

1. Michel P, Hadour G, Rodriguez C, Chiari P, Ferrera R. Evaluation of a new preservative

solution for cardiac graft during hypothermia. J Heart Lung Transplant. 2000 Nov;19(11):1089–97.

2. Michel P, Vial R, Rodriguez C, Ferrera R. A comparative study of the most widely used solutions for cardiac graft preservation during hypothermia. The Journal of Heart and Lung Transplantation. 2002 Sept 1;21(9):1030–9.
